# Ursodeoxycholic acid prevents selenite-induced oxidative stress and alleviates cataract formation: In vitro and in vivo studies

**Published:** 2012-01-18

**Authors:** Hui-Ping Qi, Shu-Qin Wei, Xiang-Chun Gao, Nan-Nan Yu, Wan-Zhen Hu, Sheng Bi, Hao Cui

**Affiliations:** 1 Department of Ophthalmology, the First Affiliated Hospital of Harbin Medical University, Harbin, China; 2 Perinatal Epidemiology, Sainte-Justine Hospital, University of Montreal, Montreal, Canada; 3 Department of Ophthalmology, the Fourth Affiliated Hospital of Harbin Medical University, Harbin, China; 4 Department of Hematology, the First Affiliated Hospital of Harbin Medical University, Harbin, China; 5 Department of Neurology, the First Affiliated Hospital of Harbin Medical University, Harbin, China; 6 Central Laboratory, the First Affiliated Hospital of Harbin Medical University, Harbin, China

## Abstract

**Objective:**

To evaluate the antioxidative and anticataractogenic potential effect of ursodeoxycholic acid (UDCA) on selenite-induced cataract in vitro and in vivo.

**Methods:**

Enucleated rat lenses were incubated in M199 medium alone (Group I), with 200 μM selenite (Group II), or with 200 μM selenite and 500 μM UDCA (Group III). Selenite was administered on the third day and UDCA treatment was from the second to the fifth day. The development of cataracts was observed under an inverted microscope. Total antioxidative capabilities (T-AOC), mean activities of superoxide dismutase (SOD), catalase (CAT), glutathione peroxidase (Gpx), glutathione reductase (GR) and glutathione S-transferase (GST), levels of reduced glutathione (GSH), malondialdehyde (MDA), and total sulfhydryl content were analyzed in lenticular samples. In vivo, cataracts were induced in 12-day-old pups by single subcutaneous injections of sodium selenite. The test groups received 180 mg/kg bodyweight/day of UDCA intraperitoneally on postpartum days 11–16 or 0.5% UDCA drops four times daily on postpartum days 11–25.

**Results:**

In vitro, morphological examination of the lenses revealed dense vacuolization and opacification in Group II, minimal vacuolization in 12.5% of Group III, and no opacification in 87.5% of Group III. In Group I, all lenses were clear. UDCA significantly (p<0.05) restored GSH and total sulfhydryl, and decreased MDA levels. T-AOC and the mean activities of the antioxidant enzymes were elevated following treatment with UDCA. In vivo, 0.5% UDCA drops resulted in only 20% nuclear cataract development and 180 mg/kg of UDCA intraperitoneally led to 50% development, compared to 100% in the control group (p<0.05).

**Conclusions:**

UDCA prevents selenite toxicity and cataractogenesis by maintaining antioxidant status and GSH, protecting the sulfhydryl group, and inhibiting lipid peroxidation in lenses.

## Introduction

Cataracts are the leading cause of irreversible blindness worldwide [1]. Over 50 million people worldwide suffer from cataracts and the number will increase as individuals in the current generation grow older [2,3]. Currently, the only cure for cataracts is surgical removal of the opaque lenses and substitution with clear ones. However, this operation is not equally available to all and an artificial lens does not have the overall optical qualities of a normal lens [4]. Preventing or delaying the onset of cataracts by pharmacological approaches may lessen this burden, reduce the occurrence of sightlessness, and enhance the quality of life for much of the world’s older, and diabetic populations [5].

Both epidemiological and experimental studies provide evidence that oxidative stress is a major mechanism in the initiation and progression of cataracts [6,7]. Accordingly, lenses have evolved antioxidant systems to defend against the toxic damage of reactive oxygen species (ROS) or free radicals, including antioxidants, such as reduced glutathione (GSH), and antioxidant enzymes such as superoxide dismutase (SOD), catalase (CAT), glutathione S-transferase (GST), and glutathione reductase/peroxidase (GR/Gpx) [8-10]. Selenite-induced cataracts have been widely used to study mechanisms of cataract formation and to screen potential anti-cataract agents [5]. Several studies [11,12] have shown positive associations between antioxidant intake and reduced incidence or progression of nuclear cataracts.

The use of bear bile has been practiced in China and other Asian countries for thousands of years [13]. China’s State Pharmacopoeia lists approximately 28 types of medicines containing bear bile, 15 of which are used in ophthalmology [14]. Ursodeoxycholic acid (UDCA) is the pharmacologically active ingredient contained in bear bile. It has been synthetized from cholic acid and approved by the health administrations of several countries for treatment of liver diseases and gallstones [15,16]. UDCA has been proved to prevent the oxidative injury induced by several agents, through a direct antioxidant effect or an increase in antioxidant defenses [17]. Perez et al. [18] found that UDCA treatment during pregnancy prevented oxidative injury in the placenta and fetal liver [19] by upregulating the activities of catalase, glutathione peroxidase, and glutathione-S-transferase, increasing GSH content and the GSH/glutathione disulfide ratio, and reducing oxidative stress-induced apoptosis. Mulhern et al. [20] showed that tauroursodeoxycholic acid (TUDCA), another form of the bile acid components, could suppress lens epithelial cell death and alleviate cataract formation in galactosemic rat lenses though alleviating endoplasmic reticulum (ER) stress. Song et al. [21] suggested that UDCA protected the chaperone activity of α-crystallin in human lens. Today, UDCA is produced and marketed by several manufacturers worldwide because of its powerful antioxidant activity, its ability to be well tolerated, and its low toxicity [15]. Furthermore, the above findings may lead to the development of UDCA as an anticataract agent. Therefore, the authors of this paper proposed that the antioxidant action of UDCA could contribute to beneficial effects in cataract patients. No previous work has studied the effects of UDCA on oxidative stress or on the activities of antioxidant enzymes in cataracts. The present study employed a selenite-induced cataractogenesis model to test the hypothesis that UDCA could retard cataract formation and modulate antioxidant status in vitro and in vivo.

## Methods

### Reagents

M-199 culture medium, fetal bovine serum (FBS) and antibiotic solution were purchased from Gibco (Grand Island, NY). Sodium selenite was obtained from Sigma Chemical Company (St. Louis, MO). UDCA was purchased from Amresco Inc. (Solon, OH). Culture plates were acquired from Corning Inc. (Corning, NY). Protein and enzyme quantification kits were obtained from Jiancheng Bioengineering Institute (Nanjing, China).

### Lens culture and grouping

Lenses were obtained from the eyes of 5-week-old male Wistar rats (Institute of Experimental Animals, Harbin Medical University) by a posterior approach under deep anesthesia. Animal care and protocols were in accordance with and approved by the Institutional Animal Ethics Committee. Lenses were cultured in M-199 medium supplemented with 20 mM HEPES, 10% heat-inactivated (56 °C for 0.5 h) fetal bovine serum (FBS), 100 U/ml penicillin, and 0.1 mg/ml streptomycin at 37 °C in a humidified atmosphere of 5% CO_2_ as described previously [22]. Lenses were placed in individual wells of 24-well culture dishes containing 1 ml medium/well for five days; those developing opacification in the first 24 h were discarded. Previous studies have shown that these culture conditions maintain lens vitality over 96 h without significant changes in cellular biochemical indexes, such as sodium pump activity and protein synthesis [22,23].

Lenses were grouped as follows with eight lenses in each group: Group I, cultured in a normal medium alone (normal control); Group II, cultured in a medium supplemented with sodium selenite (cataract-untreated); and Group III, cultured in a medium supplemented with sodium selenite and UDCA (cataract-treated). UDCA treatment (500 μM) was administered to Group III from the second to the fifth day while selenite administration (200 μM) to Group II and Group III was on the third day. Selenite sodium was dissolved in saline (150 mM NaCl) and UDCA was dissolved in a solution at pH 8.3 (NaCl 150 mM, Na_2_CO_3_ 100 mM) [24]. During and at the termination of the experiment, lenses were observed for cataract formation using an inverted microscope (at 40×, 100×, and 200×).

### Preparation of lenses for analysis

The lenses from each group were homogenized in 0.9% neutral normal sodium (w/v 1:9), and centrifuged at 2,200× g for 15 min at 4 °C. The supernatant obtained was stored at −80 °C in aliquots, pending further analysis. Protein in each sample was estimated by the Coomassie brilliant blue method, using BSA as a standard. Analysis of each antioxidant parameter was repeated three times.

### Measurement of total antioxidative capability (T-AOC)

The T-AOC was measured by the ferric reducing-antioxidant power (FRAP) assay method [25]. The ferric ion (Fe^+3^) was reduced to ferrous (Fe^+2^) form by an antioxidative substance in the supernatant, and the color that developed was detected at 520 nm with a spectrophotometer. The result was expressed as units/mg protein and one unit of T-AOC was equal to a 0.01 increase in absorbance of the reaction mixture at 520 nm per milligram protein per min under 37 °C incubation.

### Assay of antioxidant enzymes activity

#### Superoxide dismutase

Superoxide dismutase (SOD) activity in lens homogenate was assayed by using a previously reported method [26]. The degree of inhibition of 4-nitro-blue tetrazolium chloride (NBT) using the xanthine-xanthine oxidase system was measured. The change of optical density was read spectrophotometrically at 560 nm. The amount of enzyme that inhibited NBT by 50% was defined as one unit of SOD activity. Results were expressed as units/mg protein.

#### Catalase

Catalase (CAT) activity was assayed at 25 °C by the method introduced by Pedraza-Chaverrí et al. [27], based on the direct measurement of H_2_O_2_ decomposition. Researchers started the reaction by adding 20 μl of the sample to 3 ml of H_2_O_2_. The change of optical density was read spectrophotometrically at 240 nm. The decomposition of H_2_O_2_ by CAT presented in the supernatant followed a first-order kinetic reaction, as given by the equation k=2.303/t log A_0_/A_60_, where k is the first-order reaction rate constant, t is the time over which the disappearance of H_2_O_2_ was measured (60 s), and A_0_/A_60_ is the optical density at times 0 and 60 s, respectively. The result was expressed as units/g protein and one unit of CAT activity represented 1 mmol H_2_O_2_ decomposition per sec.

#### Glutathione peroxidase

The activity of glutathione peroxidase (Gpx) was determined, essentially, as described by Rotruck et al. [28]. The rate of glutathione oxidation catalyzed by the Gpx present in the supernatant, with H_2_O_2_ as a cofactor, was determined. The change in absorbance was read against a reagent blank at 412 nm on a spectrophotometer. In the present tests, the enzyme activity was expressed as units/mg protein. One unit activity was defined as the amount of enzyme that converted 1 μmol of reduced glutathione (GSH) to the oxidized form of glutathione (GSSH) in the presence of H_2_O_2_/min.

#### Glutathione reductase

The activity of glutathione reductase (GR) was assayed by the procedure of Linetsky et al. [29]. The principle of this method is that GR utilizes nicotinamide adenine dinucleotide phosphate (NADPH) to convert oxidized glutathione to its reduced form. The reaction was initiated by the addition of 20 μl of lens homogenate. The decrease in optical density was read at 340 nm for 2 min at intervals of 30 s with a spectrophotometer. The enzymatic activity was calculated using an extinction coefficient of 6.22 mM/cm for NADPH and it was expressed as mmol of NADPH oxidized/min/g protein.

#### Glutathione S-transferase

The activity of glutathione S-transferase (GST) was measured according to the procedure developed by Habig et al. [30]. The conjugation of GSH with 1 chloro and 2–4 dinitrobenzene (CDNB) was observed spectrophotometrically at 412 nm. One unit of GST was defined as the enzyme concentration required to reduce 1 μmol GSH in one min.

### Estimation of malondialdehyde content

The extent of lipid peroxidation was determined by the Bhuyan et al. method [31]. Briefly, 0.2 ml of 8.1% sodium dodecyl sulfate, 1.0 ml of 50% acetic acid, and 1.5 ml of 0.81% thiobarbituric acid aqueous solution were added in succession. Approximate 0.1 ml of the homogenate was combined with the mixture and the mixture then boiled for 80 min. The supernatant was separated after cooling by centrifugation at 4,000× g for 10 min and the intensity of the resulting pink color was read spectrophotometrically at 532 nm against a blank without tissue homogenate. Tetramethoxypropane was used as an external standard. The level of lipid peroxide was expressed as nmoles of MDA formed/mg protein.

### Estimation of reduced glutathione and total sulfhydryl content

The GSH content was estimated by the method of Hissin et al. [32]. Trichloroacetic acid was added to the lenticular homogenate and the supernatant was obtained by centrifugation of the sample. This supernatant (100 μl) was then mixed with 300 μl of 0.6 M Na_2_HPO_4_ and 100 μl 0.04% (w/v) 5, 5′-dithiobis-nitrobenzoic acid (DTNB). The resulting yellow color was read at 412 nm on a spectrophotometer. GSH standards were run simultaneously. The results were expressed in μmol/mg protein.

Briefly, the total sulfhydryl content was determined according to the description of the kit. The yellow compound given by the reaction of sulfhydryls and DTNB had a high absorption of light at 412 nm. The content of total sulfhydryl in each lens (from GSH and protein) was measured by this colorimetric method.

### In vivo studies

Wistar rat pups were divided into control (Group I), cataract-untreated (Group II), and cataract-treated (Group III and Group IV) groups. In each group (n=10), pups were housed with their mothers under standard conditions and the mothers were given normal rat chow and water ad libitum. On postnatal day 12, pups in Groups II, III, and IV received a single injection of 20 μmol/kg bodyweight sodium selenite subcutaneously, while those in Group I received only saline. One drop 0.5% UDCA was instilled every 6 h (four times daily) into the eyes of pups in the group III, starting one day before the sodium-selenite injection (on postpartum day 11), and the schedule continued for 14 days (until postpartum day 25). The 0.5% UDCA drop was at a 5 mg/ml concentration, dissolved in a solution at pH 8.3 containing NaCl 150 mM and Na_2_CO_3_ 100 mM. Pups in Group IV received intraperitoneal injections (180 mg/kg bodyweight) of UDCA on postpartum days 11–16. The incidence of cataracts was viewed by an operating microscope after dilating the pupil with 1% tropicamide on postpartum day 25.

### Statistical analysis

For the continous variables (in vitro data), the data were expressed as mean±SD, and statistics of the data used the one-way ANOVA analysis. When ANOVA results showed statistically significant differences, post hoc testing was performed for intergroup comparisons using the least significant difference (LSD) test. The χ^2^ test was applied for the categorical variables (in vivo data). A p<0.05 was considered significant. All statistical calculations were computed by the Statistical Package for Social Science (SPSS) version 11.0 (SPSS Science, Chicago, IL).

## Results

### Lens morphology in vitro

**Figure 1:**
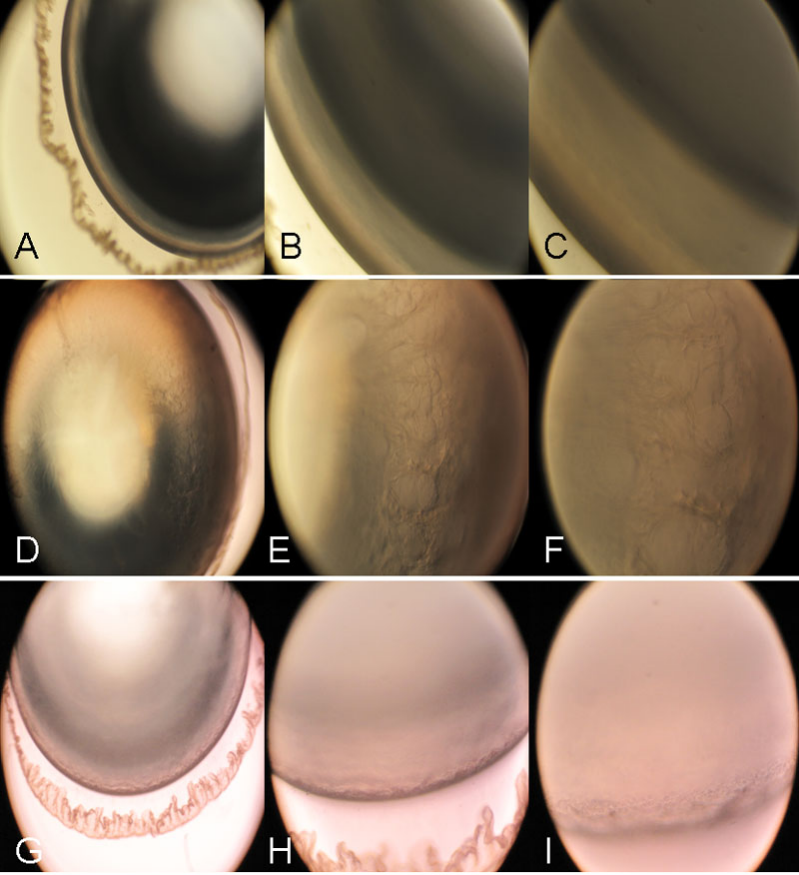
Rat lenses in various groups. **A**, **B**, and **C** are normal lenses; **D**, **E**, and **F** are selenite-induced lenses, and **G**, **H**, and **I** are selenite + UDCA-induced lenses. The magnification of **A**, **D**, and **G** is 40×; the magnification of **B**, **E**, and **H** is 100× and the magnification of **C**, **F**, and **I** is 200×.

At the termination of the experiment, all the lenses in Group I exhibited complete transparency. All the lenses in Group II exhibited dense cortical vacuolization and lenticular opacification. The majority of lenses (87.5% on average) in Group III were transparent and the remainder developed lesser amounts of cortical vacuolization when supplemented with UDCA. The results showed UDCA could prevent the formation of vacuoles and opacity to a great extent (Figure 1).

### Total antioxidative capability in lenses

**Figure 2:**
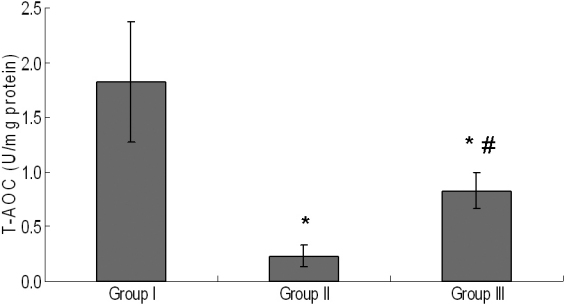
T-AOC in the lens of experimental groups. Group I: Control. Group II: Selenite-supplemented. Group III: Selenite-supplemented + UDCA treated. Values are expressed as mean±SD. Statistics of the data used one-way ANOVA followed by the least significant difference (LSD) test. * Compared with Group I: p<0.05. ^#^ Compared with Group II: p<0.05.

The T-AOC in lenses treated with selenite was significantly decreased compared to the control (p<0.05). The T-AOC in the lenses of Group III was significantly higher than that in Group II rat lenses, but it was lower than that in Group I (p<0.05; Figure 2).

### Activities of antioxidant enzymes

#### Superoxide dismutase

**Figure 3:**
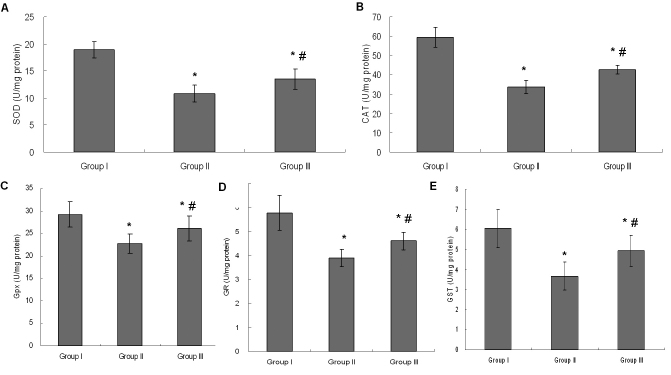
Activities of antioxidant enzymes in lenticular samples. **A**: Activity of SOD in lens. **B**: Activity of CAT in lens. **C**: Activity of Gpx in lens. **D**: Activity of GR in lens. **E**: Activity of GST in lens. Group I: Control. Group II: Selenite-supplemented. Group III: Selenite- supplemented + UDCA treated. Values are expressed as mean±SD. Statistics of the data used one-way ANOVA followed by the least significant difference (LSD) test. * Compared with Group I: p<0.05. ^#^ Compared with Group II: p<0.05.

The activity of SOD in Group II significantly decreased following selenite administration compared to the control (p<0.05), while treatment with UDCA in Group III was found to maintain significantly higher levels of SOD activity compared to Group II (p<0.05; Figure 3A).

#### Catalase

The activity of CAT in Group II significantly decreased following selenite administration compared to the control (p<0.05), while treatment with UDCA in Group III was found to maintain significantly higher levels of CAT activity compared to Group II (p<0.05; Figure 3B).

#### Glutathione peroxidase

The activity of Gpx in Group II significantly decreased following selenite administration compared to the control (p<0.05), while treatment with UDCA in Group III was found to maintain significantly higher levels of Gpx activity compared to Group II (p<0.05; Figure 3C).

#### Glutathione reductase

The activity of GR in Group II significantly decreased following selenite administration compared to the control (p<0.05), while treatment with UDCA in Group III was found to maintain significantly higher levels of GR activity compared to Group II (p<0.05; Figure 3D).

#### Glutathione S-transferase

The activity of GST in Group II significantly decreased following selenite administration compared to the control (p<0.05), while treatment with UDCA in Group III was found to maintain significantly higher levels of GST activity compared to Group II (p<0.05; Figure 3E).

### Levels of malondialdehyde in lenses

**Figure 4:**
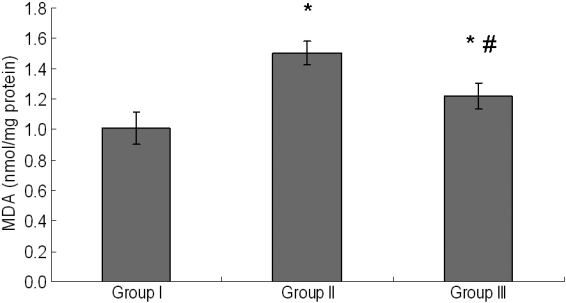
Levels of MDA (a lipid peroxidation product index) in lens. Group I: Control. Group II: Selenite-supplemented. Group III: Selenite- supplemented + UDCA treated. Values are expressed as mean±SD. Statistics of the data used one-way ANOVA followed by the least significant difference (LSD) test. * Compared with Group I: p<0.05. ^#^ Compared with Group II: p<0.05.

MDA, an indicator of lipid peroxidation, was significantly elevated following selenite induction in Group II compared to the control (p<0.05), while UDCA treatment showed a significant reduction in the levels of MDA in Group III compared to Group II (p<0.05; Figure 4).

### Levels of reduced glutathione and total sulfhydryl content in lenses

**Figure 5:**
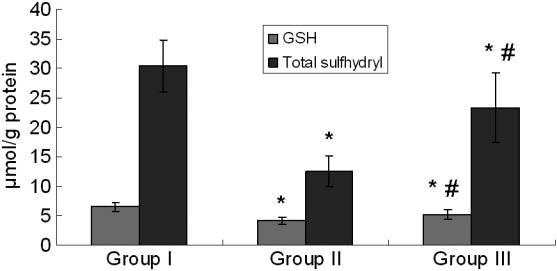
The levels of reduced GSH and total Sulfhydryl content in lens. Group I: Control. Group II: Selenite-supplemented. Group III: Selenite- supplemented + UDCA treated. Values are expressed as mean±SD. Statistics of the data used one-way ANOVA followed by the least significant difference (LSD) test. * Compared with Group I: p<0.05. ^#^ Compared with Group II: p<0.05.

Remarkable decreases in GSH levels and total sulfhydryl content were found in Group II lenses compared to the control, while GSH levels and total sulfhydryl content were elevated with UDCA treatment when compared to Group II (Figure 5).

### Effect on selenite cataract: In vivo

**Figure 6:**
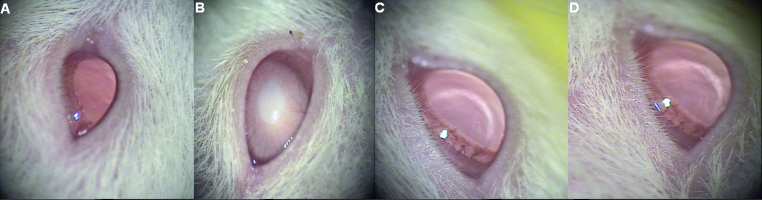
Appearance of lenses in eyes of Wistar rat pups. **A**: Lenses in normal (Group I) pup eyes: clear. **B**: Lenses in cataract-untreated (Group II) pup eyes: nuclear cataracts. **C**: Lenses in some pup eyes treated with UDCA drop (Group III): clear. **D**: Lenses in some pup eyes treated with intraperitoneal UDCA (Group IV): pinpoint opacity.

All pups that received normal saline exhibited complete transparency of the lens. Subcutaneous injections of sodium selenite led to the development of 100% nuclear opacities in the lens of Group II on postnatal day 25. In contrast, 0.5% UDCA drops led to 40% of the eyes being clear, 40% having pinpoint opacity, and only 20% developing nuclear cataracts. 180 mg/kg of intraperitoneal UDCA resulted in 20% of the eyes being clear, 30% having pinpoint opacity, and 50% developing nuclear cataracts (Table 1). Lenses in the eyes of the Wistar rat pups from different groups are demonstrated in Figure 6.

**Table 1:** Morphological examination of lenses of rat pups in the in vivo study.

**Groups**	**Clear**	**Pinpoint opacity**	**Nuclear cataract**
Group I	10 (100%)	0	0
Group II	0	0	10 (100%)
Group III	4 (40%)^*†^	4 (40%)^*†^	2 (20%)^*†^
Group IV	2 (20%)^*†^	3 (30%)^*†^	5 (50%)^*†^

Data are presented as N (%); Statistics analysis was by χ^2^ test; Group I: normal control; Group II: cataract-untreated; Group III: cataract treated with 0.5% UDCA drops; Group IV: cataract treated with 180 mg/kg bodyweight UDCA intraperitoneal injections. * Compared with Group I: p<0.05; ^†^ Compared with Group II: p<0.05.

## Discussion

Oxidative stress has been implicated in many disease processes, especially in age-related disorders, such as age-related cataracts [33]. Antioxidant systems that consist of non-enzymatic and enzymatic components protect the lens from oxidative damage and maintain lens clarity. UDCA has been proved to have antioxidant properties both in vitro [34] and in vivo [35,36]. The current study is the first to evaluate the anticataract potential of UDCA against selenite-induced cataracts. The results showed that pretreatment with UDCA could retard cataract formation and inhibit oxidative stress in the selenite cataract model, in vitro and in vivo.

Selenite-induced cataracts exhibit many general similarities to human cataracts, such as increased calcium, protein aggregation, decreased water-soluble proteins, and reduced glutathione levels [37]. They have received much attention and been the objects of model system work in the field of oxidative stress-induced cataracts. The characteristic is cortical vacuolization and opacification in vitro, while it is a nuclear cataract in the in vivo rat pup model. The underlying mechanisms of the development of both opacifications have been found to be similar [22]. Therefore, the lens opacification observed in vivo can be mimicked in vitro by the addition of Na_2_SeO_3_ to the culture medium [5]. Due to the special anatomic structure and efficient protective mechanisms in the eye, systemically and topically administered drugs have poor access to the eye [38]. To target lens successfully, the organ-cultured rat lens was used as the model to study the biochemical basis for UDCA protective functions against selenite-induced cataract in vitro. Furthermore, the UDCA protective effects were confirmed with in vivo experiments.

Alleviation of cataract formation was observed in cultured lenses with UDCA pretreatment. This observation may be attributed to the improved antioxidant status. Mitsuyoshi et al. [39] established the free-radical scavenging effects of UDCA by measuring the levels of glutathione (GSH) and thiol-containing proteins in hepatocytes; this was further confirmed by measuring MDA, TAOC, SOD, CAT, GSH-Px, GR and GST activities in this study.

Antioxidant enzymes such as SOD, CAT, and GSH-Px are present in all parts of the lens [40]. They can scavenge formed ROS. Superoxides can first be degraded into hydrogen peroxide (H_2_O_2_) by SOD, and subsequently, catalyzed into ground-state oxygen and water by catalase and enzymes of the glutathione redox cycle, including glutathione reductase (GR) and glutathione peroxidase (Gpx) [41]. Although the individual contribution of each of these enzymes in the detoxification of H_2_O_2_ is not clear, they are generally thought to work cooperatively. At low levels of H_2_O_2_, the glutathione redox cycle is responsible for protecting against H_2_O_2_-induced damage and maintaining high levels of GSH in the lens, whereas, at a higher concentration, the principal mechanism for the removal of H_2_O_2_ is catalase [42]. Many in vitro studies [43,44] have established that GR can affect cation transport systems, lens hydration, sulfhydryl groups of proteins, and membrane integrity. GST, a typical multifunctional enzyme, is also viewed as a defense mechanism against lipid peroxidation. It plays a role in the hydrophobic compounds as a thioltransferase-like redox regulator [45]. In the present study, the mean activities of SOD, CAT, Gpx, GR, and GST significantly decreased in the lenses of the cataract-untreated group (Group II), compared with normal control (Group I) rat lenses. In lenses treated with UDCA, the mean activities of antioxidant enzymes significantly increased, when compared with lenses in Group II. A similar result was found in the studies by Perez et al. [18,19], in which they demonstrated that UDCA increased the antioxidant enzyme levels in the placenta and fetal liver of rats with maternal cholestasis. The present study also assayed T-AOC levels to evaluate total antioxidative capacity: when the lenses were treated with UDCA, T-AOC levels were elevated. The results indicated that UDCA enhancement of lenticular total antioxidant capacity might include nonenzymatic and enzymatic antioxidant systems.

GSH, which is highly concentrated in the lens and helps to reduce proteins [46,47], contains a side chain of sulfhydryl (-SH) residue that enables it to protect cells against oxidants. Reduced levels of GSH have been observed in cataractous lenses [47]. The present investigation found that UDCA significantly restored the levels of GSH and total sulfhydryl (from GSH and protein) in cultured rat lenses. The present findings corroborate earlier studies where UDCA significantly restored GSH levels in isolated rat hepatocytes [40] and in rats with bile-duct-ligation-induced secondary biliary cirrhosis [36]. The enhancement of glutathione levels can be included among the beneficial effects of UDCA treatment and may be due to a higher expression of the enzymes involved in glutathione synthesis [17]. Malonedialdehyde (MDA) is a product of the breakdown of mainly unsaturated fatty acids into their essential chains through the oxidation mechanism. It is accepted as a reliable marker of the lipid peroxidation that occurs because of oxidative stress. In the present study, UDCA decreased MDA levels in the lens. These results confirmed the antioxidative effect of UDCA on selenite-administered lenses. Similar findings regarding the effect of UDCA on lipid peroxidation were reported by Ljubuncic et al. [48] in cultured macrophages and by Jüngst et al. [49] in gallbladder bile.

Selenite cataracts can be produced in suckling rats between postnatal days 10 to 14, five to six days after a single injection of selenite. The present investigation confirmed that both topical administration of 0.5% UDCA and intraperitoneal administration of UDCA 180 mg/kg into rat pups could prevent selenite-induced cataract formation. The dose of UDCA was based on the results of the pilot study. However, the incidence rate of lens opacity with UDCA treatment in vivo was higher, compared to that in vitro. The authors of this paper suspect that this may be ascribed to the transport properties of UDCA into the eyes.

In conclusion, the study showed that UDCA delayed the progression of lens opacification in selenite-induced cataracts in vitro and in vivo. UDCA may serve as an antioxidant agent, increase the levels of GSH, protect the sulfhydryl group, maintain antioxidant enzyme activities, and inhibit lipid peroxidation, thus protecting lens transparency. Therefore, research on the mechanisms of UDCA provides an important experimental base for potential drug therapies for cataracts. It remains to be determined whether UDCA is limited in its transport into the eyes. Further research is needed to evaluate the transport properties of UDCA and to enhance its ocular bioavailability.
